# Shared genetic influences do not explain the association between parent–offspring relationship quality and offspring internalizing problems: results from a Children-of-Twins study

**DOI:** 10.1017/S0033291717001908

**Published:** 2017-07-26

**Authors:** L. J. Hannigan, F. V. Rijsdijk, J. M. Ganiban, D. Reiss, E. L. Spotts, J. M. Neiderhiser, P. Lichtenstein, T. A. McAdams, T. C. Eley

**Affiliations:** 1 Institute of Psychiatry, Psychology & Neuroscience, MRC Social, Genetic & Developmental Psychiatry Centre, King's College London, London, UK; 2 Department of Psychology, The George Washington University, Washington, DC, USA; 3 Yale Child Study Center, New Haven, CT, USA; 4 Office of Behavioral and Social Science Research, NIH, Bethesda, MD, USA; 5 Department of Psychology, The Pennsylvania State University, State College, PA, USA; 6 Department of Medical Epidemiology and Biostatistics, Karolinksa Institutet, Stockholm, Sweden

**Keywords:** Behavioural genetics, children-of-twins, gene-environment correlation, internalizing problems, parent-child relationship

## Abstract

**Background:**

Associations between parenting and child outcomes are often interpreted as reflecting causal, social influences. However, such associations may be confounded by genes common to children and their biological parents. To the extent that these shared genes influence behaviours in both generations, a passive genetic mechanism may explain links between them. Here we aim to quantify the relative importance of passive genetic *v.* social mechanisms in the intergenerational association between parent–offspring relationship quality and offspring internalizing problems in adolescence.

**Methods:**

We used a Children-of-Twins (CoT) design with data from the parent-based Twin and Offspring Study of Sweden (TOSS) sample [909 adult twin pairs and their offspring; offspring mean age 15.75 (2.42) years], and the child-based Swedish Twin Study of CHild and Adolescent Development (TCHAD) sample [1120 adolescent twin pairs; mean age 13.67 (0.47) years]. A composite of parent-report measures (closeness, conflict, disagreements, expressions of affection) indexed parent–offspring relationship quality in TOSS, and offspring self-reported internalizing symptoms were assessed using the Child Behavior Checklist (CBCL) in both samples.

**Results:**

A social transmission mechanism explained the intergenerational association [*r* = 0.21 (0.16–0.25)] in our best-fitting model. A passive genetic transmission pathway was not found to be significant, indicating that parental genetic influences on parent–offspring relationship quality and offspring genetic influences on their internalizing problems were non-overlapping.

**Conclusion:**

These results indicate that this intergenerational association is a product of social interactions between children and parents, within which bidirectional effects are highly plausible. Results from genetically informative studies of parenting-related effects should be used to help refine early parenting interventions aimed at reducing risk for psychopathology.

Internalizing problems in childhood have been well established as predictors of risk for later mental health issues (Harrington *et al.* 1990; Kim-Cohen *et al.* 2003; Roza *et al.* 2003; Rutter *et al.* 2006). These problems, which include generalized symptoms of anxiety and depressed mood, are moderately heritable [see reviews by Rice *et al.* 2002 (depression); Rapee *et al.* 2009 (anxiety)] and tend to emerge early in development (Kovacs & Devlin, 1998; Kessler *et al.* 2005). As such, the possible role of parenting interactions in their aetiology has been investigated extensively, and associations have been shown with parental discipline (McKee *et al.* 2007; Laskey & Cartwright-Hatton, 2009), parental overinvolvement (Hudson & Rapee, 2001; Narusyte *et al.* 2008) and parental psychopathology (Connell & Goodman, 2002; Goodman *et al.* 2011) among many others (see meta-analytic reviews by McLeod *et al.* 2007; Yap & Jorm, 2015; Möller *et al.* 2016).

Observed associations between parenting behaviours and children's internalizing problems are often interpreted, particularly in lay contexts, as reflecting causal, parent-to-child effects, mediated via social interactions and learning processes. However, this interpretation makes assumptions about both the direction of effects and, more fundamentally, the nature of the mechanism by which these effects are mediated. Longitudinal research can help to clarify the true direction of effects, but the nature of the association often remains confounded. In particular, this is because, while a social, environmental mechanism (within which effects may still be bidirectional) is one possibility, shared genes mean that associations between biologically related parents and children may result from a *passive genetic* mechanism (or *passive gene–environment correlation*; Plomin *et al.* 1977). Children receive their autosomal DNA from both parents in approximately equal proportions – resulting in considerable (≈50%) overlap in their genes. With parents thus providing both environments *and* genes for their children, the mechanism underlying any given parent–offspring association cannot be assumed. Parenting interactions *could* be related to children's internalizing problems via the social interactions they share, but shared genes could plausibly underpin both. The coordinated use of different genetically sensitive designs is required to disentangle the roles played by these different mechanisms.

Genetically informative research designs have long provided an important tool for studying children's environmental exposures (Plomin *et al.* 1985, 1989; Plomin & Bergeman, 1991; Braungart *et al.* 1992). In such designs, the estimation of genetic effects allows environmental sources of variation to be distinguished from those that are genetically confounded (Neiderhiser, 2001). Genetically informative research indicates that parents’ behaviour towards their children is subject to influence from both parent's *and* children's genes (Neiderhiser *et al.* 2004, 2007; see reviews by Kendler & Baker, 2007; Klahr & Burt, 2014). When the objective is to estimate the extent to which an association incorporating both parent and child behaviours is affected by potential confounding by shared genes, the Children-of-Twins (CoT) design (D'Onofrio *et al.* 2003) is particularly useful (McAdams *et al.* 2014). In the CoT design, the genetically informative relationship (i.e. twinship) exists in the parent generation, with data collected from both twins and their respective offspring. The logic underlying the classical twin design is then applied to all genetic relationships within these families, to allow influences on both parent *and* child traits to be partitioned into genetic and environmental components. Moreover, the inclusion of *differential* intergenerational genetic relationships in the CoT design (an aunt in an identical twin family is more related to her nephew than an aunt in a non-identical twin family) also allows *associations* between parent and child traits to be partitioned; into passive genetic transmission (due to genes shared across the generations) and social transmission mediated through parents’ and children's interactions with one another (D'Onofrio *et al.* 2003; McAdams *et al.* 2014).

Associations between children's internalizing problems and parent characteristics have been relatively well studied using the CoT design. Primarily, these investigations have focused on associations between children's internalizing and analogous problems in the parent generation, such as anxiety and depression, finding that these associations appear to result from social, rather than passive genetic, mechanisms (Silberg *et al.* 2010; Singh & D'Onofrio, 2011; Eley *et al.* 2015; McAdams *et al.* 2015; see McAdams *et al.* 2014 for a review). Relatively, fewer CoT studies have investigated associations between parenting and children's internalizing problems. Within the Twin and Offspring Study of Sweden (TOSS) sample used in the current study, an association between maternal emotional overinvolvement and offspring internalizing problems was found to be best explained by children's genetically influenced behaviour evoking a response from the mothers (Narusyte *et al.* 2008). A later study found evidence that associations between family conflict and offspring internalizing were largely unconfounded by shared genes shared across the generations (Schermerhorn *et al.* 2011). Similarly, the link between parental criticism and adolescent somatic symptoms was found to be free of confounding via a passive genetic mechanism (Horwitz *et al.* 2015). Finally, a recent analysis of the association between closeness and affection in the parent–child relationship and adolescent self-worth found similar results; the intergenerational association was not confounded by shared genes (McAdams *et al.* 2017).

While specific parenting behaviours such as these appear to have socially mediated links with children's internalizing problems, it bears consideration that these behaviours are unlikely to be experienced by children in the way that they are commonly measured: as discrete, clearly delineated behaviours. Instead, a conceptualization of parenting as a complex amalgam of multiple behaviours may better represent the overall quality of the parent–child relationship (Belsky, 1984; Darling & Steinberg, 1993). This relationship, while influenced by specific parenting behaviours, is also shaped by the stable behavioural characteristics of both parents and children (Kendler *et al.* 1997; Kiff *et al.* 2011), reactive and situational responses (Miller *et al.* 1998; Metsäpelto *et al.* 2001; Critchley & Sanson, 2006), perceptual tendencies (Reidler & Swenson, 2012; Hannigan *et al.* 2016*a*) and development-related change (Steinberg, 2000; Ludeke *et al.* 2013; Hannigan *et al.* 2016*b*). Further, the emotional valence of this relationship in a child's life is considerable, making it a particularly relevant context in which to examine the development of internalizing problems. Assessing multiple aspects of the parent–child relationship concurrently, including adaptive as well as maladaptive behaviours, may help to provide a more comprehensive picture of a developing child's experience within their home environment. To our knowledge, no previous study has done this in a CoT framework to investigate the aetiology of links between the parent–child relationship and internalizing behaviours.

## The current study

In the current study, we apply the CoT design to data on parents’ relationship with their adolescent offspring and offspring internalizing problems, with the aim of identifying the extent of passive genetic *v.* social effects in the association between them. Genetic and environmental influences on parent–offspring relationship quality are estimated by comparing the similarity of identical and non-identical adult twins (TOSS sample) in terms of their relationship with their adolescent children. Variance in offspring internalizing problems is similarly decomposed, by comparing the phenotypic similarity of cousins in identical and non-identical twin families (TOSS) and of child twins in a parallel sample (the Swedish Twin Study of CHild and Adolescent Development, TCHAD). The extent of passive genetic *v.* social transmission is estimated by comparing avuncular (aunt/uncle with niece/nephew) correlations between parent-offspring relationship quality and offspring internalizing in the families of the identical and non-identical adult twins.

## Methods

### Samples

#### Twin and Offspring Study of Sweden (TOSS)

Data drawn from the TOSS sample, comprised 909 pairs of (same-sex) twins, their child and spouse. Twin parents in the study sample were 63% female, while their offspring were 48% female. The number of monozygotic (MZ) twins in the study sample was 765 (44.1%); included dizygotic (DZ) twins numbered 969. The average age of twins was 44.87 years (s.d. = 4.86) and of their offspring was 15.75 years (s.d. = 2.42). The TOSS sample has been described in detail elsewhere (Neiderhiser & Lichtenstein, 2008).

#### Swedish Twin Study of CHild and Adolescent Development (TCHAD)

Data drawn from TCHAD comprised 1120 adolescent twin pairs. Twins were 52% female, with an average age of 13.67 years (s.d. = 0.47), while 895 (40.2%) were MZ twins and 1334 were DZ twins. DZ twin pairs in this sample included opposite sex twin pairs (comprising 782 individuals). Full details of the TCHAD sample are provided in Lichtenstein *et al.* (2007).

In both studies, informed consent was obtained prior to individuals’ participation and both the TOSS and TCHAD projects received ethical approval from the Institutional Review Boards of the home institutions concerned (Lichtenstein *et al.* 2007; Neiderhiser *et al.* 2007).

### Measures

#### Parent–offspring relationship quality

A composite scale indexing the overall quality of the parent–offspring relationship was derived from both maternal and paternal reports on three parenting-related scales. We opted to use a composite score because of the breadth of the construct of interest – the parent–offspring relationship – and in order to make our coverage of this construct as comprehensive as possible. Scales were selected for inclusion in the composite because they specifically addressed facets of parent–offspring interactions that are relevant to child outcomes. Disagreements about rules and behaviour in the household were assessed using a 38-item Child Rearing Issues: Parent-Child Agreement scale (Hetherington & Clingempeel, 1992). Parents reported on how often certain behavioural interactions occur with seven-point Likert scale responses to items such as ‘How often have you not agreed with your child concerning if he/she uses alcohol?’. A 22-item Expressions of Affection scale (Hetherington & Clingempeel, 1992) measured both the frequency of parents’ expressions of affection towards their child and their engagement in behaviours, such as playing music together. A seven-point Likert scale was again used to record parents’ responses, and scores on this variable were reversed for inclusion in the composite. Finally, the 27-item Parent–Child Relationship (PCR) questionnaire (Hetherington & Clingempeel, 1992) was used to elicit parents’ perceptions of the closeness (items reverse-scored) and conflict in their relationship with their children (e.g. ‘How well do you and your child understand each other?’), via a five-point Likert scale.

Individual scale scores were standardized to give each an equal weight in the composite, with an overall mean taken (Cronbach's *α* = 0.68). This meant that the highest scores would represent the lowest quality parent–child relationships (characterized by high levels of disagreement and conflict and by low levels of expressed affection and closeness). Individuals were required to have some data available on at least two of the scales to receive a score on the composite and thus be included in the analyses. Composite scale scores approximated a normal distribution, and so standardized raw scores were used in the analyses. This approach of creating a composite score from multiple parenting measures reduces single-measure biases and has been used in previous analyses with similar data (Neiderhiser *et al.* 2004; Marceau *et al.* 2013).

#### Internalizing problems

Offspring internalizing problems were assessed using the Youth Self-Report version of the widely-used Child Behavior Checklist (CBCL; Achenbach & Edelbrock, 1983; Achenbach, 1991) in both samples. Self-reports (rather than parent reports) of offspring internalizing problems were used to ensure that the decomposition of the intergenerational association was not confounded by rater-specific biases. The CBCL internalizing sub-scale indexes anxious/depressed behaviours, withdrawn behaviours and somatic complaints. Responses are given on a three-point scale. Scores on this measure were positively skewed in both samples, so were transformed using a Box–Cox transformation (Cox *et al.* 1964; Sakia, 1992) prior to analysis, which resulted in approximately normal distributions. Internal consistency for this scale was good in both TOSS (Cronbach's *α* = 0.86) and TCHAD (0.88).

### Genetic analysis

#### The CoT design

**Fig. 1.**
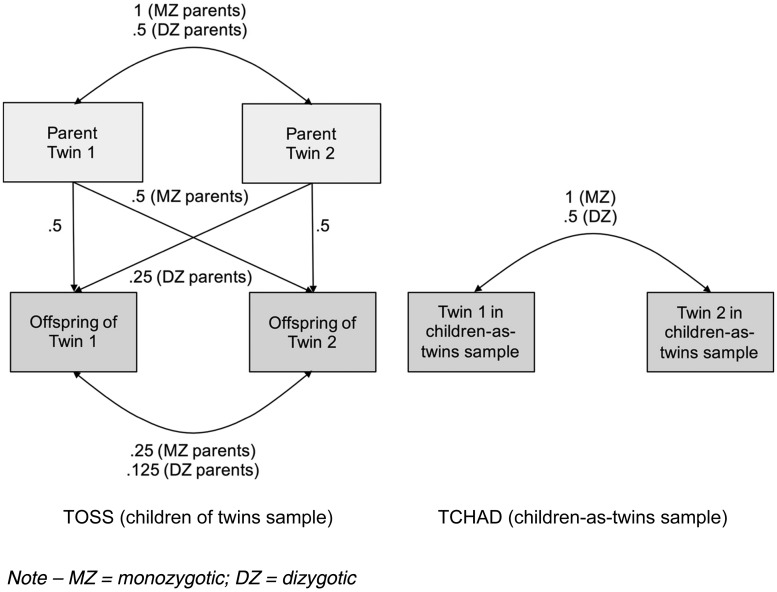
Model of the genetic correlations within the Children of Twins (TOSS) and parallel children-as-twins (TCHAD) samples.

The CoT design is an extension of the logic of the classical twin design (Rijsdijk & Sham, 2002). Its power lies in the nature of the avuncular relationship between the child of a twin and their parent's co-twin (their aunt or uncle). In the case of MZ twin-pair parents, a child will share as much genetic material with his aunt or uncle as with his own parent; whereas when the parent and aunt/uncle of the child are DZ twins, the avuncular genetic correlations are half as strong (Fig. 1). If passive genetic transmission between the parenting and child outcome phenotypes is in effect, MZ avuncular *phenotypic* correlations will therefore be higher than DZ avuncular phenotypic correlations.

**Fig. 2.**
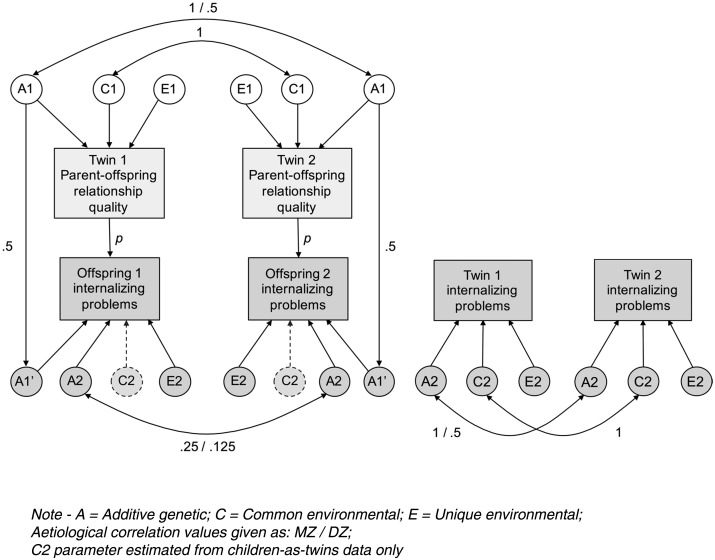
Children-of-Twins model for parent–offspring relationship quality and offspring internalizing problems, incorporating internalizing problems data from children-as-twins sample.

The full CoT model used in this study is shown in Fig. 2. Variance in the observed phenotypes is decomposed into genetic (*A*), shared environmental (*C*) and unique environmental (*E*) influences. Passive *genetic* transmission is possible via the *A*1–*A*1′ path, which is fixed at 0.5 to reflect the proportion of segregating genetic material shared, on average, by parents and children. Social transmission is possible via the central *p* path, which, although conventionally drawn as a single-headed arrow from parent to child, includes bidirectional effects.

Data from a parallel child-based sample (TCHAD) were incorporated into the part of the CoT model estimating the genetic and environmental components of variance for offspring internalizing problems. This has two main advantages. The first is an increase in power to detect genetic effects in the offspring generation. In the CoT sample, cousins in MZ families share approximately 25% of their segregating genes, whereas those in DZ families share approximately 12.5%. In a child-based sample, this ratio remains consistent with respect to genetic relatedness, but overall proportions of shared genes in the offspring generation are increased (e.g. 100% and 50% in MZ and DZ families), which increases statistical power. The second is that, in a CoT model based on a children-of-twins sample (e.g. TOSS) *only*, influences from the shared rearing environment cannot be estimated in the offspring generation, which consists of (non-cohabiting) cousins in MZ and DZ twin families. However, with the addition of data from a parallel child-based sample (TCHAD) wherein twin children do cohabit, this effect *can* be estimated (represented by ‘*C*2’ in Fig. 2). It should be noted that this strategy forms part of the Extended Children-of-Twins (ECoT) model (Narusyte *et al.* 2008), which combines children-of-twin and child-as-twin datasets in order to estimate bidirectional effects, but that our model is still specified as a standard CoT due to the unavailability of relevant parent–offspring relationship variables in the child-based sample, which is required for the ECoT specification.

## Results

Correlations within and between parents and offspring in MZ and DZ families, respectively, are presented in Table 1. MZ twin correlations in the parent generation (in the TOSS sample only) exceeded DZ twin correlations (0.35 *v.* 0.19), indicating genetic influence on parent–offspring relationship quality. In the offspring generation, cross-cousin correlations in the TOSS sample were slightly larger in MZ families compared with DZ families (0.16 *v.* 0.10), as were cross-twin correlations in the child-based TCHAD sample (0.55 *v.* 0.27), which indicates some genetic influence on offspring internalizing problems. Parent–offspring correlations, which were constrained to be equal across zygosity groups as an assumption of the model, indicated a modest but significant intergenerational association (0.21). MZ avuncular correlations were not significantly different from DZ avuncular correlations (0.07 *v.* 0.11), indicating a negligible role for passive genetic transmission effects in underpinning this association.

**Table 1.** Correlations between parent-reported parent-offspring relationship quality and offspring self-reported internalizing problems in monozygotic and dizygotic twin-parent families (TOSS) and child-as-twin families (TCHAD)

	TOSS		TCHAD	
	MZ	DZ	MZ	DZ
Parent generation				
Twin (RQ–RQ)	**0.35**	**0.19**		
*95% CI*	*0.25–0.44*	*0.11–0.27*		
Child generation				
Cousin (IP–IP)	**0.16**	0.10		
*95% CI*	*0.06–0.26*	*0.00–0.19*		
Twin (IP–IP)			**0.55**	**0.27**
*95% CI*			*0.49–0.60*	*0.25–0.33*
Intergenerational				
Parent–offspring (RQ–IP)	**0.21**	**0.21**		
*95% CI*	*0.16–0.26*	*0.16–0.26*		
Avuncular (RQ–IP)	0.07	**0.11**		
*95% CI*	*0.00–0.15*	*0.05–0.17*		

Bold typeface signifies statistical significance; all confidence intervals should be italicized. RQ, parent–offspring relationship quality; IP, internalizing problems.

Parent–offspring correlations are constrained to be equal across twin order and zygosity, in line with theoretical expectations that they will not differ significantly (the validity of this assumption was affirmed by constraining the saturated model and observing no significant decrement of model fit).

The results of the model-fitting procedure are presented in Table 2. First, we compared the fit of the full CoT model to the saturated phenotypic model, in order to test the basic assumptions of the model. Subsequent model comparisons were between the full CoT model and a series of reduced sub-models. All sub-models were nested in the larger full model, meaning that model fit could be compared using a χ^2^ difference test of their −2LL values. Sub-models 1–3 tested the significance of shared environmental (*C*) parameters in both generations (independently and then together), as these were either non-significant or estimated at zero in the full model. None of these models resulted in a decrement of fit, so the most parsimonious (model 3) was used as a basis for further constraints. We ran models 4–6 to investigate the characteristics of the association between parent–offspring relationship quality and offspring internalizing problems. Model 4, in which the *A*1′ genetic transmission parameter was fixed to zero, provided the best fit for the data. This indicates that passive genetic effects did not contribute significantly to the association. In contrast, both model 5 (in which the genetic transmission path was reinstated and social transmission was fixed to zero) and model 6 (which tested the overall significance of the intergenerational association) resulted in a significantly poorer fit to the data. Below, we present and describe the results of model 4 (the best-fitting model overall) alongside the full model for comparison.

**Table 2.** Fit indices from model-fitting of parent-reported parent–offspring relationship quality and offspring self-reported internalizing problems

	ep	−2LL	df	AIC	ΔLL	Δdf	*p*
Model							
Saturated model	38	15 574.94	5586	4402.94	–	–	–
Full CoT model	11	15 608.30	5613	4382.30	33.35	27	0.19
Sub-models							
1. Drop *C*1	10	15 608.61	5614	4380.61	0.32	1	0.57
2. Drop *C*2	10	15 608.30	5614	4380.30	0.00	1	1
3. Drop *C*	9	15 608.61	5615	4378.61	0.32	2	0.85
**4. Direct transmission only (drop *C*; *A*1′**)	**8**	**15 609.70**	**5616**	**4377.70**	**1.40**	**3**	0.70
5. Genetic transmission only (drop *C*; *p*)	8	15 620.39	5616	4388.39	12.09	3	0.01
6. No intergenerational association (drop *C*; *p*; *A*1′)	7	15 681.53	5617	4447.53	73.24	4	<0.001

ep, estimated parameters; −2LL, log likelihood; df, degrees of freedom; AIC, Akaike's Information Criterion.

Best-fitting model in bold typeface; nested models compared with full model using formal test of change in −2LL value; CoT refers to full Children-of-Twins model incorporating child-as-twin data.

**Fig. 3.**
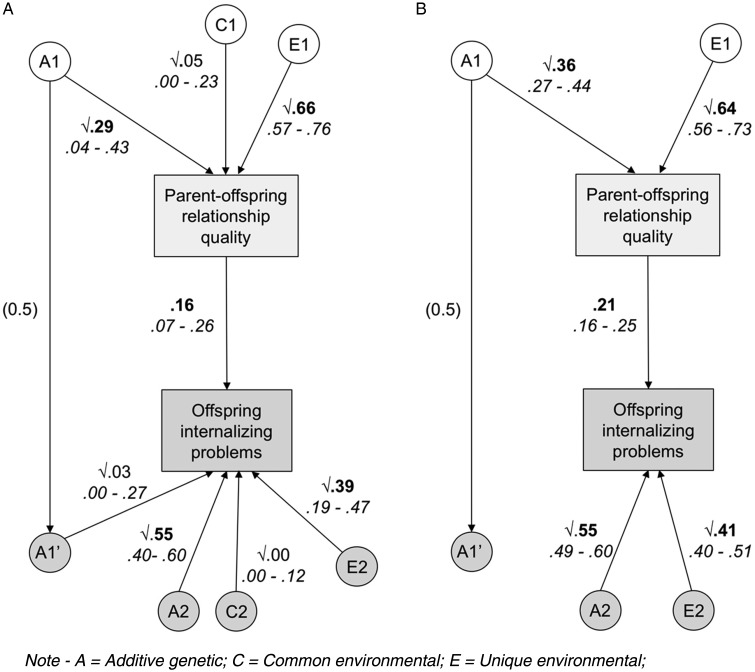
Path diagram showing parameter estimates from the full (A) and best-fitting reduced (B) model of parent-reported parent–offspring relationship quality and offspring self-reported internalizing problems.

Figure 3 presents the estimates from the full (panel A) and best-fitting models (panel B) of parent-reported parent–offspring relationship quality and offspring self-reported internalizing problems. The 95% confidence intervals (CIs) are presented below each estimate in italicized typeface. All non-significant parameters in the full model are dropped (as described above) in the best-fitting model, leaving the final estimate of the effects of social transmission at 0.21. The heritability of parent–offspring relationship quality is estimated at 0.36, and the heritability of offspring internalizing problems at 0.55. Shared environmental influences were not significant in either generation, with non-shared environmental influences explaining the remaining variance in parent–offspring relationship quality (0.64) and offspring internalizing problems (0.41), respectively.

## Discussion

Associations between measures of parenting and child outcomes are not straightforward to interpret, due to the inter-relatedness of genetic and environmental influences within the home environment (Collins *et al.* 2000; Rutter, 2004; Horwitz & Neiderhiser, 2015). In the current study, we used a CoT design, to show that an intergenerational, passive genetic pathway does not explain a significant proportion of the association between parent–offspring relationship quality and offspring internalizing. Instead, the best-fitting model involved only social transmission effects. Although the direction and precise nature of the social effects operating to underpin the intergenerational association cannot be determined using this study design, these results add to a growing body of genetically informed parenting research that has implications for the study and treatment of childhood internalizing problems.

### Parent–offspring relationship quality and offspring internalizing problems not related via passive genetic effects

The primary goal of this study was to test whether parent–offspring relationship quality, as reported on by parents, was related to offspring self-reported internalizing problems via passive genetic transmission. Evidence of such a process would mean that the same genetic factors that influenced parenting in one generation also influenced internalizing problems in the other. Our model-fitting results strongly suggested that this is not the case within our CoT sample: passive genetic effects did not explain intergenerational association to any significant extent.

Though limited in number, previous studies investigating the aetiology of intergenerational associations between parenting phenotypes and internalizing problems have produced similar findings (Lynch *et al.* 2006; Narusyte *et al.* 2008; Schermerhorn *et al.* 2011; McAdams *et al.* 2014, 2017; Horwitz *et al.* 2015). To further contextualize this finding, it is worth emphasizing that our sample did meet three important theoretical pre-conditions for passive genetic transmission to be a plausible mechanism. First, consistent with findings from previous studies (see reviews by Kendler & Baker, 2007; Klahr & Burt, 2014), there were genetic influences on parents’ perceptions of their relationship with their child. Second, similarly, adolescent children's internalizing problems were subject to genetic influence. This, too, is in line with the existing evidence base (Rice *et al.* 2002; Rapee *et al.* 2009; Polderman *et al.* 2015). Finally, we saw evidence of a significant intergenerational association between the measures. The magnitude of this association (0.21) was in line with results from previous meta-analyses of intergenerational associations between parenting and child internalizing problems more generally (McLeod *et al.* 2007; Yap & Jorm, 2015; Möller *et al.* 2016). In this context, our finding of no role for a passive genetic mechanism appears unlikely to be related to any unusual characteristics of either the sample or the measures used in our study.

### Evidence for the importance of social interactions; bidirectional effects possible

With no evidence of passive genetic transmission in the association between parent–offspring relationship quality and offspring internalizing problems, a social mechanism was found to be the best explanation of this relationship within our models. Significantly, this was the first CoT study to investigate the nature of this kind of association using a broad measure of the parent–offspring relationship, rather than specific parenting behaviours, such as discipline or overinvolvement. Our finding seems to reduce the possibility that the specificity of the parenting phenotypes used in earlier studies precluded the detection of passive genetic confounding that *was* present in the broader parent–child relationship. Instead, the importance of social interactions for producing such associations seems relatively unequivocal from studies using this design. However, there are several plausible alternatives for the precise mechanisms involved.

The first possible mechanism involves parent-to-child effects, in which poor quality parent–offspring interactions act as an environmental risk factor for children to develop internalizing problems. Evidence of such a mechanism has been shown for some parenting phenotypes and child behavioural outcomes (Asbury *et al.* 2003; Burt *et al.* 2005; Bornovalova *et al.* 2014) though it is notable that there is some debate, prompted by limited evidence for sustained shared environmental influences on behaviour later in development and into adulthood, about the enduring effects of parenting as experienced similarly by siblings (summarized in Burt, 2014) other than as a result of extremely maladaptive parental behaviours (e.g. abuse and neglect; Norman *et al.* 2012).

A second possible mechanism involves the reverse direction of causation, with child internalizing problems influencing parent–offspring relationship quality. Within this model of child-to-parent effects, it is important to note that, with parents and children sharing only ≈50% of segregating genes, *child* genes may still play a role in influencing the parent–offspring relationship (via their influence on the child's internalizing problems). Indeed, evidence from a range of study designs has demonstrated the role of children's genetically influenced behaviour in evoking parenting responses (Avinun & Knafo, 2014; Klahr & Burt, 2014). Given the strength of this previous evidence and the consistency of this mechanism with longstanding ‘bidirectional effects’ models of family functioning (Bell, 1979; Bronfenbrenner, 1986), it seems likely that some genetically influenced child-driven effects underpin this association. Although the extent of this *evocative* mode of gene–environment correlation cannot be estimated in this study, we can conclude that any child genes influencing parent–offspring relationship quality in this way are *not* the same as those genes influencing the relationship via the parents’ behaviour; otherwise, their effect would have resulted in significant direct genetic transmission in the models.

The possible influence of children's genes in the social transmission path in the CoT model is the reason that we avoid referring to it with any variant of the term ‘environmental’, which could reasonably be assumed to mean ‘entirely non-genetic’. Instead, we label this mode of transmission with a term that emphasizes what is common to the two mechanisms outlined above: namely, a requirement for *social* interaction. Evidence from adoption studies is broadly supportive of this, with small or no genetic transmission effects found between birth parent characteristics and their adopted-away children's emotional outcomes (Kerr *et al.* 2013; McAdams *et al.* 2015). Adoption designs offer a strong and direct test of the relative importance of parents’ provision of genes *v.* their provision of environments, but are often limited in scale and representativeness. In addition to CoT samples, novel designs leveraging differences in family types created by assisted conception (Harold *et al.* 2011) or divorce and remarriage (Kendler *et al.* 2015, 2016) offer promise of progress in this area.

Ruling out confounding by passive genetic transmission, quantifying the importance of social interactions and understanding the direction of effects within associations between parenting and child psychopathology are important for appropriately targeting family-based interventions. To rigourously define targets for interventions within the family environment, experimental work must be coordinated with findings from studies that are designed to isolate and delineate specific mechanisms and risk processes (Howe *et al.* 2010; Brody *et al.* 2013). Recent evidence suggests that findings from molecular genetic studies are becoming increasingly applicable in this regard (Brody *et al.* 2015; Keers *et al.* 2016). As well as being important for designing effective interventions and targeting them efficiently, precisely defining mechanisms by which risk for psychopathology is mediated in families is crucial for improving our understanding of how and why problems such as depression emerge when they do. In the short term, approaches to the treatment of internalizing problems in childhood and adolescence should continue to account for links with children's family environments, which remain robust even when controlling for genetic relatedness. Evidence from the current study suggests that the social features of the parent–child relationship should be considered as part of the clinical picture for internalizing problems well into adolescence.

### Limitations

The limitations and main assumptions of the CoT approach have been discussed in detail elsewhere (D'Onofrio *et al.* 2003; McAdams *et al.* 2014). The CoT formulation of the equal environments assumption that underpins twin research in general is that offspring in MZ families have no more avuncular contact (time spent with aunts/uncles) than offspring in DZ families. Where studied, this assumption has been shown to hold adequately to support these analyses (Koenig *et al.* 2010).

A specific issue in the current study concerns the nature of parents’ reporting on the quality of the parent–offspring relationship, which may only partly reflect their child's subjective experience (Hannigan *et al.* 2016*a*). To the extent that parents’ own internalizing problems influenced their reporting, and were influenced by the same genes as child internalizing problems, this would inflate the estimate of genetic transmission in the model. However, two factors mitigate this limitation. The first is that previous studies have detected no significant genetic transmission when examining parent–adolescent associations for anxiety (Eley *et al.* 2015) or depression (McAdams *et al.* 2015). Secondly, by using offspring self-reports of their internalizing problems, we avoid the potential for spurious genetic overlap caused by a genetically influenced rater effect.

A final limitation concerns the decision not to separate maternal and paternal reports of the parent–offspring relationship. Some previous work has indicated that different modes of mechanisms may underpin intergenerational transmission via maternal and paternal behaviours (Narusyte *et al.* 2011). However, we opted not to split our sample on this basis for due to power concerns, and a lack of a strong rationale for expecting such differences. Nonetheless, other studies could investigate this possibility in future.

## Conclusion

The findings from this study add to a growing body of literature highlighting the importance of social transmission in associations between parenting-related phenotypes and offspring internalizing problems. Although other designs are required to quantify the extent of bidirectionality in this phenotypic transmission, two clear conclusions can be drawn from the results of these analyses. First, shared genes do not appear to contribute to overlap between the quality of the parent–offspring relationship and offspring internalizing problems. Second, the intergenerational transmission that does occur is thus contingent on parents’ and children's exposure to one another. Both of these conclusions are non-trivial and have implications for developmental scientists and clinicians alike. Developing an appropriately nuanced understanding of the interactions and behavioural cycles that produce and maintain the associations between parenting interactions and child outcomes relies upon the continued investigation of potential sources of genetic confounding.
